# Muscle fibre size optimisation provides flexibility for energy budgeting in calorie-restricted coho salmon transgenic for growth hormone

**DOI:** 10.1242/jeb.107664

**Published:** 2014-10-01

**Authors:** Ian A. Johnston, Daniel Garcia de la serrana, Robert H. Devlin

**Affiliations:** 1 Scottish Oceans Institute, School of Biology, University of St Andrews, St Andrews, KY16 8LB, UK; 2 Fisheries and Oceans Canada, 4160 Marine Drive, West Vancouver, BC V7V 1N6, Canada

**Keywords:** Growth, Myotube formation, Transgenesis, Optimal fibre size hypothesis

## Abstract

Coho salmon (*Oncorhynchus kisutch*) transgenic for growth hormone (GH) show substantially faster growth than wild-type (WT) fish. We fed GH-transgenic salmon either to satiation (1 year; TF) or the same smaller ration of wild-type fish (2 years; TR), resulting in groups matched for body size to WT salmon. The myotomes of TF and WT fish had the same number and size distribution of muscle fibres, indicating a twofold higher rate of fibre recruitment in the *GH* transgenics. Unexpectedly, calorie restriction was found to decrease the rate of fibre production in transgenics, resulting in a 20% increase in average fibre size and reduced costs of ionic homeostasis. Genes for myotube formation were downregulated in TR relative to TF and WT fish. We suggest that muscle fibre size optimisation allows the reallocation of energy from maintenance to locomotion, explaining the observation that calorie-restricted transgenics grow at the same rate as WT fish whilst exhibiting markedly higher foraging activity.

## INTRODUCTION

Growth hormone (GH) has pervasive effects on growth rate, behaviour, feeding, metabolism, osmoregulation, smoltification and immunity in fish (Björnsson, 1997). Growth rate is substantially higher in *GH*-transgenic salmonid fish than in wild-type (WT) fish, although the effects vary with promoter type and family origin (Leggatt et al., 2012). Fast growth in transgenics is associated with markedly higher appetite and feeding motivation than WT fish (Sundstrom et al., 2003).

GH binds to receptors in the liver and induces insulin-like growth factor-1 (IGF1) synthesis, which is secreted into the general circulation, promoting growth in peripheral tissues such as skeletal muscle (Johnston et al., 2011). Binding of IGF1 to its receptor activates several downstream signaling cascades including the PI3K-Akt-TOR pathway to stimulate protein synthesis and inhibit protein degradation by the ubiquitin proteasome pathway (Johnston et al., 2011).

Primary effects of *GH* transgenesis can be distinguished from secondary effects linked to appetite by comparing transgenics fed either to satiation (TF) or with the reduced ration (TR) of WT fish, producing groups of the same body size. *GH*-transgenic coho salmon [*Oncorhyncus kisutch* (Walbaum 1792)] fed to satiation had higher plasma GH and increased levels of tissue GH relative to WT fish (Raven et al., 2008). Plasma GH levels were higher still in TR fish, whereas plasma and tissue IGF1 concentrations were similar in TR and WT groups, reflecting similar feeding and growth rates (Raven et al., 2008). TR fish also maintain a much higher feeding motivation and are more active than WT fish (Sundstrom et al., 2003).

Teleosts recruit muscle fibres until they reach approximately 40–50% of the maximum adult size (Johnston et al., 2011). One report suggested that muscle in *GH*-transgenic coho salmon grew more by hyperplasia than hypertrophy than in WT fish (Hill et al., 2000), although in this study fibre number was not estimated. In order to test the hypothesis that *GH* transgenesis enhances hyperplastic muscle growth, we compared fibre number and diameter in WT, TF and TR groups of the same body length and measured expression of *gh, igf1* and genes required for myotube formation.

## RESULTS AND DISCUSSION

**Fig. 1.**
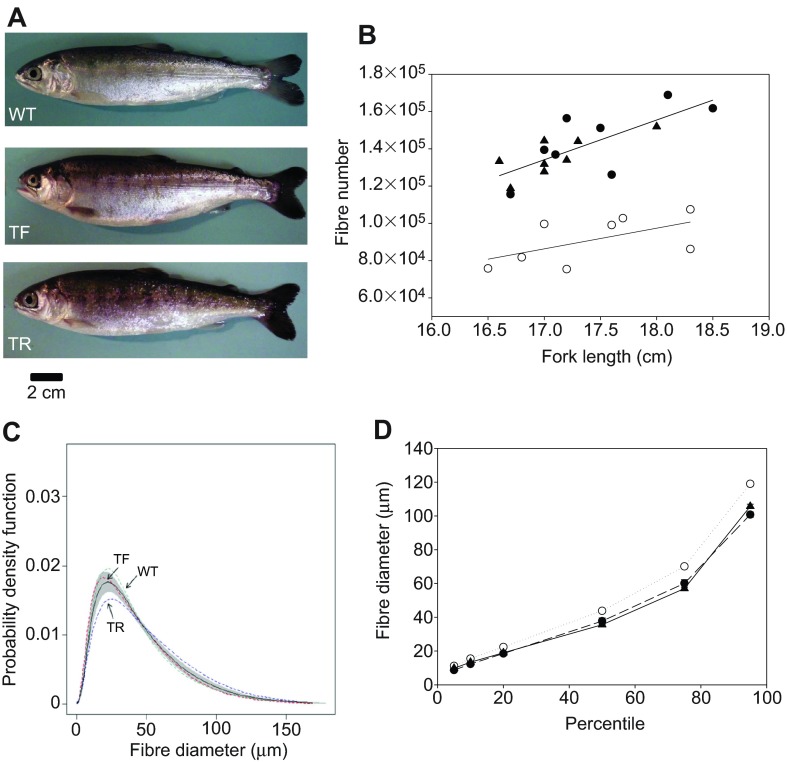
**Analysis of muscle growth patterns in coho salmon (*Oncorhynchus kisutch*).** (A) Representative images of fish in the different treatment groups. WT, wild type; TF, growth hormone (GH) transgenics fed to satiation; TR, calorie-restricted GH transgenics growing at the WT rate. (B) Relationship between fibre number and fish length. Filled triangles, WT; filled circles, TF; open circles, TR. Lines were fitted by least squares regression. (C) Probability density functions (PDFs) of muscle fibre diameter. The dashed lines represent the average PDFs of groups and the solid line the PDF of the combined groups. The shaded area represents the 1000 bootstraps of the combined group. (D) Percentiles of fibre diameter for WT, TF and TR groups. Values represent means ± s.e.m.

**Fig. 2.**
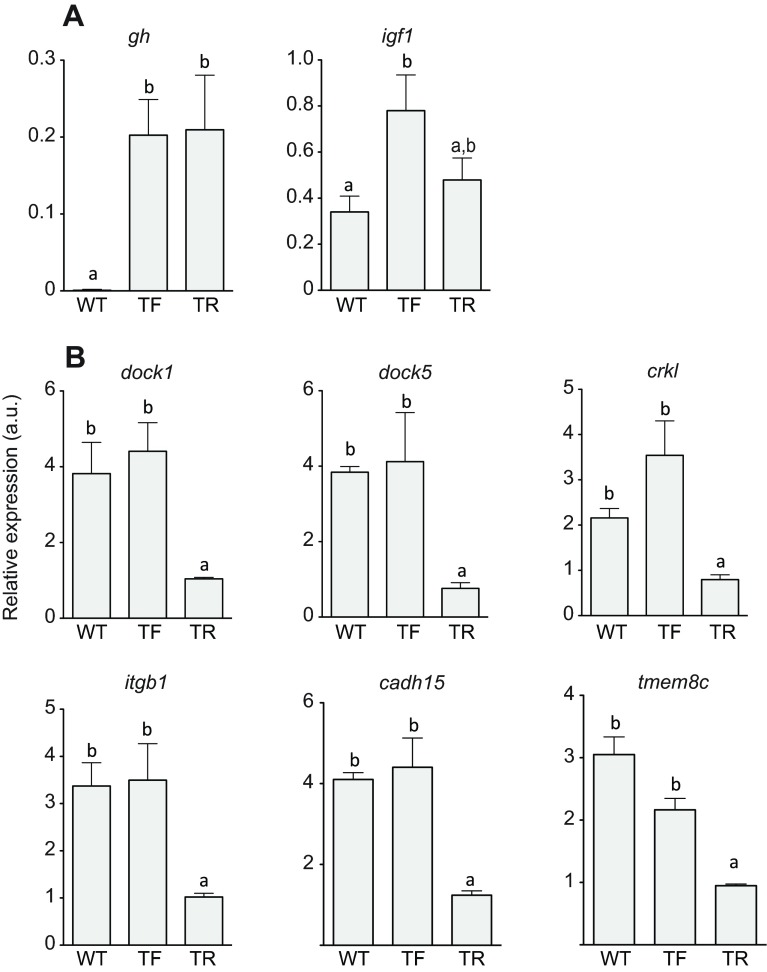
**Gene expression analysis in coho salmon (*Oncorhynchus kisutch*) fast skeletal muscle.** Gene expression in fast skeletal muscle measured by qPCR for *gh*, *igf1*, *dock5*, *dock1*, *cadherin-15*, *tmem8c*, *itgb1* and *crkl* (see supplementary material Table S2 for abbreviations). (A) GH axis genes. (B) Myotube formation genes. Results represent means ± s.e.m., 6 fish per group. Different letters indicate significant differences between means (*P*<0.05).

TF coho salmon had a higher appetite and grew faster than WT fish, recruiting fast muscle fibres at twice the rate, but showed a similar contribution of hyperplasia and hypertrophy to reach a given body length, resulting in a similar average muscle fibre size (Fig. 1A,B; supplementary material Table S1); i.e. the hypothesis of an increased importance of hyperplasia in these transgenics was not supported (Hill et al., 2000). Unexpectedly, TR fish recruited 49% fewer fibres than WT fish on the same feeding regime and 59% fewer fibres than TF fish (*P*<0.05; Fig. 1B; supplementary material Table S1). Because there were only relatively small and not statistically significant differences in total muscle cross-sectional area between groups (supplementary material Table S1), this result reflects a greater contribution of fibre hypertrophy to growth in TR fish. The average fibre diameter was 20% greater in TR fish (49 μm) than in the TF and WT groups (41 μm; supplementary material Table S1). We fitted probability density functions (PDFs) to the distributions of fibre diameter and compared specific percentiles using multi-Wilcoxon tests. The average PDF of the TR group was significantly different from that of the TF and WT groups (Kolmogorov–Smirnoff; *P*<0.05). TR fish had larger diameter fibres across the whole range of fibre sizes, i.e. increased hypertrophy was evident for cohorts of fibres with different birthdays (Fig. 1C,D). Growth hormone (*gh*) mRNA levels (Fig. 2A) were similar in TF and TR fish and much higher than in WT fish, as expected (*P*<0.01). In contrast, *igf1* expression in muscle was significantly higher in TF than in WT fish (*P*<0.05), reflecting higher food intake and growth (Sundstrom et al., 2003), whereas WT and TR fish were not

significantly different from each other. The expression of genes required for myoblast fusion during hyperplastic growth, including *dock1, dock5, crkl, itgb1, cadh15* and *tmem8c*, were twofold to fourfold downregulated in TR relative to the other groups (*P*<0.05; gene names are given in full in supplementary material Table S2).

Muscle fibre size is under strong evolutionary selection in fish and can be adjusted by altering the lifetime production of muscle fibres (Johnston et al., 2003). ATP-dependent ion pumping counteracts passive ion leak across the sarcolemmal membrane so that the energy cost of maintaining a negative resting membrane potential is proportional to fibre surface/volume ratio. Thus large fibres are cheaper to maintain than smaller ones (Johnston et al., 2012; Jimenez et al., 2013). The optimal muscle fibre size hypothesis envisages a trade-off between minimising surface/volume ratio on the one hand and avoiding diffusional constraints for diffusion of gas and metabolites on the other (Johnston et al., 2012, Johnston et al., 2003). Examples of fibre size optimisation over evolutionary time scales include the radiation of Antarctic notothenioid fishes following climatic cooling, resulting in a relaxation of diffusional constraints and ‘giant muscle fibres’ through a dramatic reduction in body-size-corrected fibre number (Johnston et al., 2003). Similarly, dwarfism in land-locked salmonid and stickleback populations was associated with a large reduction in fibre number relative to the large-bodied ancestral anadromous state, enabling a similar scaling of fibre diameter to body size (Johnston et al., 2012). The results of the present study indicate that, at least under certain circumstances, fibre size optimization may also operate within the lifetime of an individual.

The GH-axis is tightly coupled to the energy status of the fish and linked to feed intake via the expression of GH, IGF1 and their receptors (Raven et al., 2008). TR fish maintained a high GH output whilst experiencing a limitation in the supply of the amino acids and other nutrients required for growth. Our working hypothesis is that the uncoupling of the GH-axis from energy status directly affected some as-yet-unknown component of the signalling pathways regulating myotube formation and hypertrophic growth, providing the stimulus for muscle fibre size optimization. GH transgenics driven by the metallothionein-B promoter showed increased GH expression in all non-pituitary cell types including muscle and were not sensitive to the metabolic signals normally influencing endogenous GH regulation, e.g. GH receptor expression was not increased with calorie restriction in TR fish as it was in WT fish (Raven et al., 2008). It is therefore possible that the effects of calorie restriction on fibre production differ between TR and WT fish, but this remains to be investigated. We found that the median fibre diameter was 20% higher in the TR than the WT fish (Fig. 1B), which is expected to produce proportional energy savings in costs of ionic homeostasis. The energy saved could theoretically be reallocated to other aspects of the energy budget such as routine swimming activity, and may provide an explanation as to why TR fish were observed to exhibit much higher foraging activity whilst growing at a rate similar to that of WT fish fed the same diet.

## MATERIALS AND METHODS

### Fish

Coho salmon (*Oncorhynchus kisutch*) were reared in a containment facility at Fisheries and Oceans Canada, West Vancouver. WT fish were from the 2010 brood of Chehalis River strain (BC, Canada). The strain M77 transgenic coho salmon were derived from the Chehalis River strain produced using the OnMTGH1 construct as previously described (Leggatt et al., 2012).

Fish were reared in freshwater at 10±1°C with a natural photoperiod, and fed a commercial diet (Skretting, Vancouver, Canada). The 2011 brood of GH transgenics were fed to satiation thrice daily (TF group). The 2010 GH transgenics (TR group) were fed the same ration as the WT group, to produce similar body masses (supplementary material Table S1). Experiments were conducted meeting Canadian Council for Animal Care guidelines, and were approved by the Department of Fisheries and Oceans Pacific Region Animal Care Committee under Animal Use Protocol no. 10-016. TF, TR and WT groups showed no significant differences in fork length (*L*_f_); however, body mass (*M*) was significantly higher in the TF group than in the WT group (*P*<0.05; supplementary material Table S1) and condition factor (*M*/*L*_f_^3^×100) was higher in the TF group than either the TR or WT groups, reflecting differences in body shape (Fig. 1A). The total cross-sectional area of fast muscle at the position of the anal vent was not statistically different between groups (supplementary material Table S1).

### Muscle morphometry

A steak ~5 mm thick was made at the level of the anal vent. Three blocks were prepared to sample one entire half of the myotomal cross-section, and these were frozen in isopentane (2-methyl butane) cooled to its freezing point in liquid nitrogen. Sections were stained with myosin ATPase to distinguish between fibre types and hematoxylin and eosin for morphometric analysis (Johnston et al., 2012). Photographs of tissue sections were taken at a magnification of ×200, and four fields, selected at random, were photographed per block per fish. The cross-sectional areas of the entire fast muscle portion of the myotome and approximately 300 fast muscle fibres per block were digitised. In total, 800 fast muscle fibres were randomly selected per fish using a program written in R+ (http://www.r-project.org/) and smooth PDFs were fitted using a kernel function. The average smoothing parameter used (0.284) was similar between groups. Bootstrap techniques (*n*=1000) were used to distinguish underlying structure in the distributions from random variation. A non-parametric Kolmogorov–Smirnoff two-sample test was used to test the null hypothesis that the PDFs of groups were equal over all diameters (see Johnston et al., 2012). Data on fish size and muscle cellularity parameters were tested for normality (Shapiro–Wilk test) and equal variance and analysed using a one-way ANOVA with pairwise multiple comparisons by the Holm–Sidak method (overall significance level equal to 0.05).

### Gene expression analysis

Pure fast muscle was dissected from dorsal epaxial myotomes. Total RNA extraction, quality analysis and concentration protocols were as described previously (Macqueen et al., 2013). Tissues were sampled at a similar time of day for six fish per group and the RNA was stored at −80°C. A detailed description of the cDNA synthesis, primer design, qPCR reaction setup and data analysis is provided elsewhere (Macqueen et al., 2013) and in supplementary material Table S2. Briefly, a total of 1 μg of RNA from six individuals for each of the treatments (WT, TF, TR) was reverse transcribed to cDNA using the Quantitec reverse transcription kit (QIAGEN, Manchester, UK) including a gDNA removal step. Minus reverse transcriptase was performed using 1 μg of RNA from a pool created with RNA from all samples. Six microlitres per sample were mixed with 7.5 μl of SensiFAST SYBR Lo-ROX 2X master mix (Bioline, London, UK) containing 400 nmol l^−1^ of sense/antisense primers. Reactions were performed in duplicate in a Mx3005P Thermocycler (Agilent, Berkshire, UK), with one cycle of 2 min at 95°C and 40 cycles of 5 s at 95°C and 20 s at 65°C, followed by a dissociation curve analysis, which resulted in a single peak in all cases. The stability of the four housekeeping genes *rpl27, rpl13, ef1a* and β-*actin* was analysed as previously described (Macqueen et al., 2013). *Rpl13* and *ef1a* were found to be the most stable reference genes (average expression stability value M=0.058). Normalization of gene expression was performed using the geometric average of *rpl13* and *ef1a*. All expression values are expressed as arbitrary units. Expression between groups was compared using a one-way ANOVA with a Bonferroni *post hoc* correction using the SPSS21 statistical package (IBM).

## Supplementary Material

Supplementary Material
