# Supplementary material for: Muscle fibre size optimisation provides flexibility for energy budgeting in calorie-restricted coho salmon transgenic for growth hormone
Source: J Exp Biol. 2014 Oct 1;217(19):3392–5. doi: 10.1242/jeb.107664 (PMC4182283; doi:10.1242/jeb.107664)
Supplement: Supplementary Material [file supp_217_19_3392__index.html]

Muscle fibre size optimisation provides flexibility for energy budgeting in calorie-restricted coho salmon transgenic for growth hormone — Supplementary Material 

# Muscle fibre size optimisation provides flexibility for energy budgeting in calorie-restricted coho salmon transgenic for growth hormone

## JEB107664 Supplementary Material

**Files in this Data Supplement:**

- **Supplementary Material**
