# Supplementary material for: Muscle fibre size optimisation provides flexibility for energy budgeting in calorie-restricted coho salmon transgenic for growth hormone
Source: J Exp Biol. 2014 Oct 1;217(19):3392–5. doi: 10.1242/jeb.107664 (PMC4182283; doi:10.1242/jeb.107664)
Supplement: Supplementary Material [file supp_217.19.3392_JEB107664.pdf]

Table S1. Body size and muscle cellularity parameters for Coho salmon. TF: transgenics fed to satiation; TR: transgenics fed the same ration and growing at the same rate as wild type (WT) salmon. FL: fork length; TCA: total area of fast muscle per myotomal cross-section; FD: fibre diameter; FN: number of fibres per myotomal cross-section. Values represent Mean ± SE, n= 8 fish per group.

Group	Mass (g)	FL (cm)	TCA (mm^2)	FD (μm)	FN
TF	68.4 ± 2.9	17.5 ± 0.2	253.4 ± 9.8	41.0 ± 0.8	144,500 ± 6500
TR	63.5 ± 3.1	17.4 ± 0.2	225.0 ± 9.5	49.1 ± 0.4	91,000 ± 4500
WT	57.7 ± 2.0	17.1 ± 0.	248.0 ± 9.9	41.3 ± 0.5	135,700 ± 8900

Table S2. Primer sequences and characteristics

	Gene symbol	Forward	Reverse	Product size (bp)	Efficiency (E%)	Tm (C^o)
dedicator of cytokinesis protein 1	*dock1*	AGGCAAGATGGATGATGAGG	AGTTTCAGGGTCACCCACAG	150	94.5	87.5
dedicator of cytokinesis protein 5	*dock5*	CTACGCCCACTACATCAGCA	CGCAGAAACACACCAATCTG	149	93	83.6
crk-like protein	*crkl*	ACACGGAGTGTTTTTGGTGAG	GTCCCCTATGCGGAACTGT	160	102	86
cadherin15 (m-cadherin)	*cad15*	TCCTGAGTGCTGTGGATGAG	TTCCCCTGTCTCCAGCTCTA	152	99.8	86.5
myomaker	*tmem8c*	AGCCTACATTGCCAAGATGC	TCACACGCATGGTAAATCGT	147	99.5	87.5
integrin beta 1	*itgb1*	GGATTACCCCATCGACCTCT	TCAACGAAGGAACCAAAACC	150	100.5	83
growth hormone	*gh*	TGAGACCAATCGACAAGCAG	TGATGCCCACTTTGTGGTTA	180	97.6	84.5
insulin-like growth factor 1	*igf1*	ATGTACTGTGCCCCTGTCAAG	CTTGTCTGGGTGCTGTGCT	150	96.5	88
60S ribosomal protein l13	*rpl13*	AAAGAGTACCGCTCCAAGCTC	CTGGCCTTCTCCTTCTTGTG	149	99.8	87
60S ribosomal protein l27	*rpl27*	CCAGGTCTCCTGTCGATCAT	CATAGATGGGCACTGTGTGG	140	98.2	88.5
elongation factor 1 alpha	*ef1a*	GCAAGAACGACCCTCCAAT	CGGTCGATCTTCTCCTTGAG	157	96.8	89
beta actin	*ßactn*	CCAGGTCTCCTGTCGATCAT	CATAGATGGGCACTGTGTGG	151	100.7	86

Tm: Melting temperature
